# *Lacticaseibacillus rhamnosus* ATCC 53103 and *Limosilactobacillus reuteri* ATCC 53608 Synergistically Boost Butyrate Levels upon Tributyrin Administration Ex Vivo

**DOI:** 10.3390/ijms24065859

**Published:** 2023-03-20

**Authors:** Pieter Van den Abbeele, Mallory Goggans, Stef Deyaert, Aurélien Baudot, Michiel Van de Vliet, Marta Calatayud Arroyo, Michael Lelah

**Affiliations:** 1 Cryptobiotix SA, Technologiepark-Zwijnaarde 82, 9052 Ghent, Belgium; 2 NutriScience Innovations, 130C Old Gate Lane, Milford, CT 06460, USA; 3 Laboratory of Microbiology, Ghent University, Karel Lodewijk Ledeganckstraat 35, 9000 Ghent, Belgium; 4 Institute of Agrochemistry and Food Technology (IATA), Spanish Research Council (CSIC), Carrer del Catedràtic Agustín Escardino Benlloch, 7, 46980 Valencia, Spain

**Keywords:** gut microbiota, ex vivo, probiotic, prebiotic, synbiotic, tributyrin, *Lacticaseibacillus rhamnosus* LGG, *Limosilactobacillus reuteri*, butyrate, SIFR^®^

## Abstract

Modulation of the gut microbiota is a trending strategy to improve health. While butyrate has been identified as a key health-related microbial metabolite, managing its supply to the host remains challenging. Therefore, this study investigated the potential to manage butyrate supply via tributyrin oil supplementation (TB; glycerol with three butyrate molecules) using the ex vivo SIFR^®^ (Systemic Intestinal Fermentation Research) technology, a highly reproducible, in vivo predictive gut model that accurately preserves in vivo-derived microbiota and enables addressing interpersonal differences. Dosing 1 g TB/L significantly increased butyrate with 4.1 (±0.3) mM, corresponding with 83 ± 6% of the theoretical butyrate content of TB. Interestingly, co-administration of *Limosilactobacillus reuteri* ATCC 53608 (REU) and *Lacticaseibacillus rhamnosus* ATCC 53103 (LGG) markedly enhanced butyrate to levels that exceeded the theoretical butyrate content of TB (138 ± 11% for REU; 126 ± 8% for LGG). Both TB + REU and TB + LGG stimulated *Coprococcus catus*, a lactate-utilizing, butyrate-producing species. The stimulation of *C. catus* with TB + REU was remarkably consistent across the six human adults tested. It is hypothesized that LGG and REU ferment the glycerol backbone of TB to produce lactate, a precursor of butyrate. TB + REU also significantly stimulated the butyrate-producing *Eubacterium rectale* and *Gemmiger formicilis* and promoted microbial diversity. The more potent effects of REU could be due to its ability to convert glycerol to reuterin, an antimicrobial compound. Overall, both the direct butyrate release from TB and the additional butyrate production via REU/LGG-mediated cross-feeding were highly consistent. This contrasts with the large interpersonal differences in butyrate production that are often observed upon prebiotic treatment. Combining TB with LGG and especially REU is thus a promising strategy to consistently supply butyrate to the host, potentially resulting in more predictable health benefits.

## 1. Introduction

The human colon is colonized by trillions of microorganisms that strongly influence health. Alterations of the gut microbiota have been associated with disease conditions such as metabolic and immune disorders, liver dysfunctions, inflammatory bowel disease, colorectal cancer, cardiovascular conditions, and central nervous system disorders [1,2,3,4,5,6]. A key role of the gut microbiota is to ferment indigestible glycans, which results in the production of short-chain fatty acids (SCFA), among other metabolites. SCFAs have cornerstone functions in maintaining gut homeostasis but also affect extra-intestinal systems [7,8,9,10]. Butyrate is of special relevance to health as it exerts multiple functions such as acting as a histone deacetylase (HDAC) inhibitor or signaling through G protein-coupled receptors (GPCRs) [11]. Butyrate signaling involves the control over central pathways regulating gut homeostasis, including the inhibition of NF-κB activation or IFN-γ signaling, the upregulation of PPARγ, and control over cell proliferation, differentiation, and apoptosis [12]. Butyrate is also the main energy source for colonocytes and promotes barrier integrity by modulating tight junctions and mucus production [11,13,14,15,16,17]. In addition, butyrate metabolism by the epithelium consumes local oxygen and stabilizes the hypoxia-inducible factor (HIF), a transcription factor influencing barrier protection and immune response [18]. Increasing butyrate levels is thus a promising strategy to maintain metabolic homeostasis, intestinal barrier integrity, and balanced immune responses.

A first strategy to increase butyrate supply to the host could consider the administration of butyrate-producing bacteria. Key butyrate-producing species of the human gut belong to the *Lachnospiraceae* and *Ruminococcaceae* families and produce butyrate via butyrate kinase (e.g., *Coprococcus eutactus* and *Subdoligranulum variabile*) or butyryl CoA:acetate CoA transferase (e.g., *Eubacterium rectale, Anaerobutyricum hallii, Coprococcus catus,* and *Faecalibacterium prausnitzii*) [19], with specific species being able to convert acetate and lactate to butyrate in a process called cross-feeding [20]. The species capable of producing butyrate are thus very different from the traditional probiotics that are often lactic acid bacteria belonging to the *Lactobacillaceae* and *Bifidobacteriaceae* families. Some butyrate-producing species have been described to comply with probiotic criteria [21,22], but the commercialization of novel strict anaerobic microorganisms is a complex and expensive task, due to both regulatory and practical constraints (e.g., production, storage, and in vivo delivery of viable amounts). A second strategy to increase butyrate supply therefore considers increasing fiber intake. Fibers can increase butyrate production by stimulating specific butyrate producers in situ. However, a substantial interindividual variability in butyrate production upon fiber intake has been observed [23], resulting in unpredictable outcomes of interventions. Finally, while oral butyrate supplementation has been shown to improve clinical symptoms of inflammatory bowel disease, insulin sensitivity, diabetic inflammation, and an intestinal barrier, administering butyrate can be challenging for several reasons, including short metabolic half-life, toxicity, and patient intolerance [24]. Overall, while the health benefits of butyrate are generally acknowledged, its supply to the host remains difficult to manage in vivo.

Preclinical studies have the potential to complement clinical studies as they allow for reducing the impact of confounding factors affecting the gut microbiota, including diet, environment, or host genetics. Further, they allow insights to be obtained on the production of metabolites such as SCFA that are rapidly absorbed in vivo. There is however increasing awareness of a so-called ‘Valley of Death’ between preclinical and clinical research [25]. A major challenge in preclinical gut microbiome research is the drastic compositional alteration of in vivo-derived microbiota, used to inoculate preclinical models, to form in vitro-adapted microbiota. This in vitro bias is highly pronounced for short-term models where fast-growing, aerotolerant taxa represent >50% of the communities within 24 h [26,27,28,29]. Similarly, the current generation of long-term gut models impose very defined nutritional and environmental conditions, thus enriching taxa that thrive under these specific conditions [30,31], within three days after inoculation [32]. A second drawback of many models is the low throughput, often resulting in less robust study designs, lacking parallel controls and/or replicates. It is, however, of key importance to include biological replicates to acknowledge that interpersonal differences are the main driver of differences in the human microbiota, largely exceeding differences between lumen/mucus or differences along colonic regions [33]. The relevance of interpersonal differences also follows from the observation that they impact the outcome of interventions [34].

This study investigated whether glyceryl tributyrate (tributyrin; TB) could enhance butyrate levels in an ex vivo simulated colonic environment when dosed as such and in combination with *Limosilactobacillus reuteri* ATCC 53608 (REU) or *Lacticaseibacillus rhamnosus* ATCC 53103 (LGG). TB is a triglyceride, i.e., a glycerol esterified with butyrate at the 1, 2, and 3 positions [24]. The rationale for combining TB with REU/LGG is that the REU/LGG could ferment glycerol derived from the hydrolysis of TB (to one glycerol and three butyrate molecules) and in doing so produce lactic acid, a precursor of butyrate production, thus providing a dual mode of action to boost butyrate levels. Further, both strains are of particular interest, as on the one hand, LGG is the most documented probiotic strain [35], while REU has been demonstrated to convert glycerol to reuterin, an antimicrobial compound [36], thus providing an additional unique mechanism for microbiota modulation upon tributyrin (and thus also glycerol) intake. The research question was addressed using the ex vivo SIFR^®^ (Systemic Intestinal Fermentation Research) technology [37]. The SIFR^®^ technology is a highly reproducible gut model that accurately preserves in vivo-derived microbiota throughout the entire duration of the experiment. Most importantly, using three structurally different carbohydrates, it was demonstrated that findings within 24–48 h in the SIFR^®^ technology (down to species level) are predictive for findings of clinical studies where such carbohydrates are repeatedly administered over 2–6 weeks. Owing to its high throughput, the SIFR^®^ technology also enables addressing interpersonal differences. In the current study, samples from six human adults were included to evaluate the consistency of ex vivo treatment effects of TB, REU, LGG and combinations thereof. In the current study TB, REU, and LGG were thus incubated ex vivo as described below and not administered directly to the human volunteers.

## 2. Results

### 2.1. Microbiota of Study Subjects Covered Interpersonal Differences in Gut Microbiota Composition

**Figure 1:**
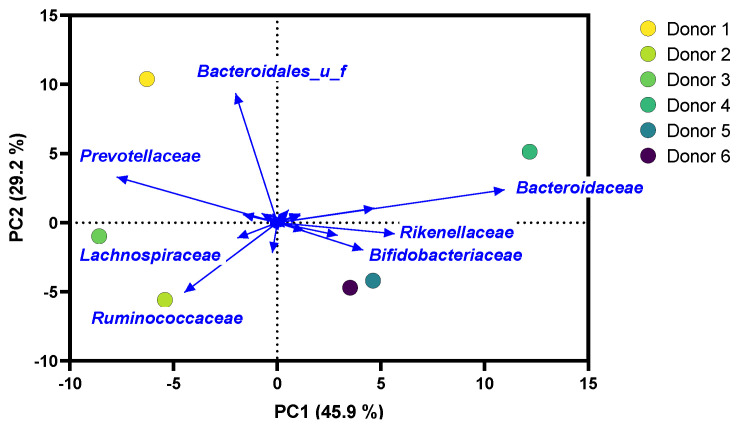
**Distinct interpersonal differences in microbial composition were observed for the 6 human adults**. Principal component analysis (PCA) summarizing microbial composition at family level. The PCA was based on centered abundances (%).

A PCA at the family level of the fecal microbiota of the six human adults used during the current study demonstrated that there were marked interpersonal differences in microbiota composition at baseline, mainly driven by different levels of *Prevotellaceae, Ruminococcaceae,* and *Bacteroidaceae* (Figure 1), which have previously been identified as key families to stratify the human gut microbiota according to the concept of enterotypes [38]. Samples of donors 1, 2, and 3 were positioned to the left side of the PCA due to high *Prevotellaceae* levels (7.2–11.9%) (Figure S1). Further, the microbiota of donors 2 and 3 had high *Ruminococcaceae* levels (21.1–22.4%), while high *Bacteroidales*_u_f levels (16.5%) were noted for donor 1. Samples of donors 4, 5, and 6 were positioned to the right side of the PCA, due to remarkably high *Bacteroidaceae* levels for donor 4 (19.6%), high *Bifidobacteriaceae* levels for donor 5 (11.5%), and *Rikenellaceae* levels of around 10% for donors 4 and 6.

### 2.2. REU and LGG Remained Viable throughout the Entire Duration of the 48 h Ex Vivo Experiment

**Figure 2:**
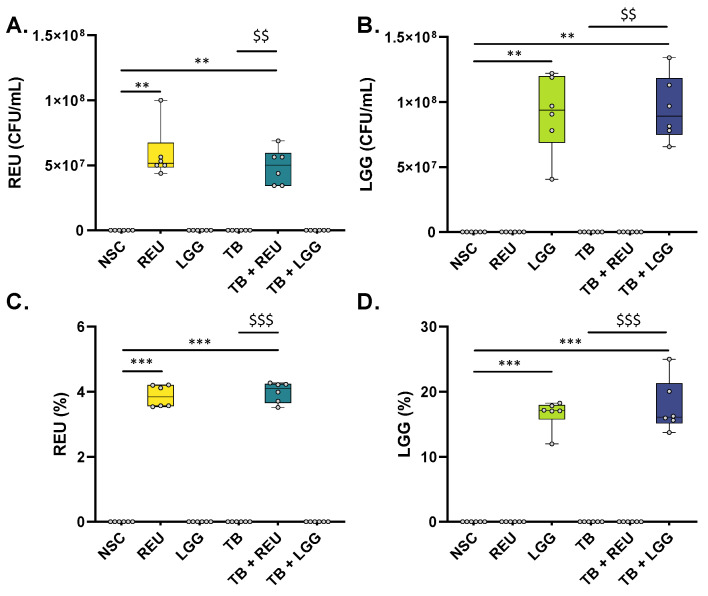
**REU and LGG were exclusively detected in study arms where they were dosed, while remaining viable along the entire duration of the 48 h ex vivo experiment**. Levels of REU (**A**,**C**) or LGG (**B**,**D**), after 48 h of incubation in the presence of fecal microbiota of 6 human adults using the ex vivo SIFR^®^ technology, as detected via culture-dependent selective enumeration (CFU/mL; **A**,**B**) or culture-independent shallow-shotgun sequencing (%; **C**,**D**). Statistical differences compared to the NSC are indicated with * (0.01 < *p*_adjusted_ < 0.05), ** (0.001 < *p*_adjusted_ < 0.01) or *** (*p*_adjusted_ < 0.001), while differences between TB + REU/TB + LGG and TB are indicated with $/$$/$$$. REU = *Limosilactobacillus reuteri*; LGG = *Lacticaseibacillus rhamnosus*.

Upon inoculation, selective enumeration of the *Lactobacillaceae species* at 0 h showed that REU and LGG were, respectively, inoculated at 4.1 ± 0.5 × 10^7^ CFU/mL and 8.7 ± 1.5 × 10^7^ CFU/mL. After 48 h of incubation, LGG and REU were exclusively detected in reactors where they were inoculated, both by culture-independent (shallow shotgun sequencing; Figure 2A,B) and by culture-dependent techniques (LAMVAB agar; Figure 2B,D). This confirms the selectivity of both detection methods and suggests that LGG and REU were not present in the fecal samples of the tested donors prior to inoculation. Interestingly, viable levels of LGG and REU (CFU/mL) were similar or even slightly increased at 48 h compared to 0 h (on average +5% for LGG and +43% for REU), thus demonstrating that the SIFR^®^ technology provided optimal conditions for LGG and REU to remain viable over the entire duration of the 48 h incubation. As a remark, LGG and REU levels were not impacted by the presence of TB.

### 2.3. TB Increased Butyrate Levels, Which Were Further Enhanced upon Probiotic Co-Administration

**Figure 3:**
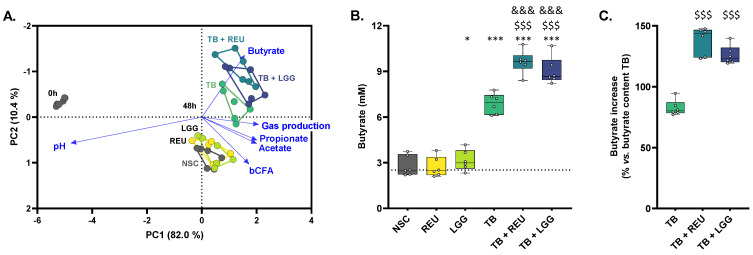
**TB increased butyrate levels, which were further enhanced upon REU/LGG co-supplementation**. (**A**) Principal component analysis (PCA) biplot summarizing the impact of two *Lactobacillaceae species* (LGG and REU), tributyrin oil (TB), and combinations thereof on markers of microbial activity, compared to an untreated NSC, at 0 h (INO) and after 48 h of colonic incubations for human adults (n = 6). (**B**) Butyrate levels (mM) and (**C**) proportional butyrate increase compared to the NSC, versus the theoretical butyrate content of TB (1 g TB/L = 4.96 mM butyrate). The line in the box plot is shown at the median value in the NSC. Statistical differences compared to the NSC are indicated with * (0.01 < *p*_adjusted_ < 0.05), ** (0.001 < *p*_adjusted_ < 0.01) or *** (*p*_adjusted_ < 0.001), while differences between TB + REU/TB + LGG and TB are indicated with $$$ (*p*_adjusted_ < 0.001). Differences between TB + REU/TB + LGG and the respective probiotic (REU/LGG) are indicated with &&& (*p*_adjusted_ < 0.001). bCFA = branched fatty acids; NSC = no substrate control; REU = *Limosilactobacillus reuteri*; LGG = *Lacticaseibacillus rhamnosus*.

A PCA based on fundamental fermentation parameters (pH, gas production, SCFA, bCFA) (Figure 3A) provided comprehensive insight into overall treatment effects as the first two components explained 92.4% of the variation of the dataset. There was a marked differential clustering of 0 h and 48 h samples, reflecting the strong production of acetate, propionate, butyrate, bCFA, and gasses between 0 and 48 h. The expansion of a complex gut microbiota over the duration of the experiment is a core aspect of the ex vivo SIFR^®^ technology [37].

At 48 h, there was a marked differential positioning of TB-treated samples compared to the NSC, upwards along PC2. Butyrate levels were primarily responsible for this TB-mediated effect as it significantly increased from, on average, 2.8 mM in the NSC up to 6.9 mM (TB) (Figure 3B). As the theoretical butyrate content of 1 g/L TB is 4.96 mM, a recovery of, on average, 83% was obtained upon TB treatment (Figure 3C). Remarkably, while the *Lactobacillaceae species* as such did not impact butyrate levels (except for a minor increase with LGG), butyrate increased up to 9.6 mM (TB + REU) and 9.0 mM (TB + LGG) for the combinations of TB with the *Lactobacillaceae species*. Butyrate recoveries largely exceeded 100% of the butyrate content in TB and reached values as high as 138% and 126% for TB + REU and TB + LGG, respectively, suggesting that glycerol fermentation could have contributed to a further butyrate increase. As the theoretical glycerol content of 1 g TB is 1.65 mmol glycerol, assuming a 1:1 glycerol to lactate/pyruvate conversion and 4:3 lactate to butyrate conversion [20], this would yield 1.24 mmol of butyrate or thus increase butyrate levels up to 125% of the theoretical butyrate content of TB. While observed butyrate levels are in this range for TB + LGG (126%), butyrate levels even increased further for TB + REU (138%), suggesting a remarkable synergistic effect of TB and REU on butyrate. Interestingly, the synergistic effects of combinations of TB with REU/LGG were highly consistent across the six human adults tested. Finally, acetate, propionate, valerate, and bCFA levels were not affected or only mildly affected by the different treatments (Figure S2).

### 2.4. The Combination of TB with REU Increased Microbial Diversity

**Figure 4:**
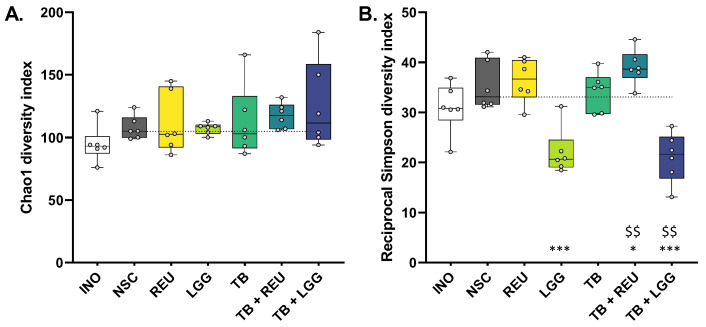
**Microbial diversity was maintained in the SIFR^®^ technology (from INO to NSC), with TB + REU further increasing diversity**. Impact of two *Lactobacillaceae species* (LGG and REU), tributyrin oil (TB), and combinations thereof, on the (**A**) Chao1 diversity index and (**B**) the reciprocal Simpson diversity index (n = 6). Samples were collected at 0 h (INO) and after 48 h of simulated colonic incubations. The line in the box plot is shown at the median value in the NSC. Statistical differences compared to the NSC are indicated with * (0.10 < *p*_adjusted_ < 0.20), ** (0.05 < *p*_adjusted_ < 0.10) or *** (*p*_adjusted_ < 0.05), while differences between TB + REU/TB + LGG and TB are indicated with $$ (0.05 < *p*_adjusted_ < 0.10). REU = *Limosilactobacillus reuteri*; LGG = *Lacticaseibacillus rhamnosus*.

First, both diversity indices suggested a similar or even higher diversity for NSC incubations at 48 h as compared to the original inocula (INO), confirming that the ex vivo SIFR^®^ technology allows for the growth of a broad range of in vivo-derived gut microbes (Figure 4). This was further substantiated by the sustained similarity between the microbiota of the inoculum and the NSC at 48 h (Figure S3).

Further, none of the treatments significantly affected estimated species richness, as measured by the Chao 1 diversity index, except for a tendency to higher richness for TB + REU (p_non-adjusted_ = 0.20) (Figure 4A). In contrast, microbial diversity in terms of both species’ richness and evenness (reciprocal Simpson diversity index) was strongly affected in specific conditions (Figure 4B). LGG treatment lowered species evenness, reflecting the presence of high LGG levels. In contrast, REU as such maintained species evenness, while TB + REU even significantly increased microbial diversity in terms of species evenness.

### 2.5. The Combination of TB with LGG and Especially REU Increased the Abundance of Specific Butyrate-Producing Species

**Figure 5:**
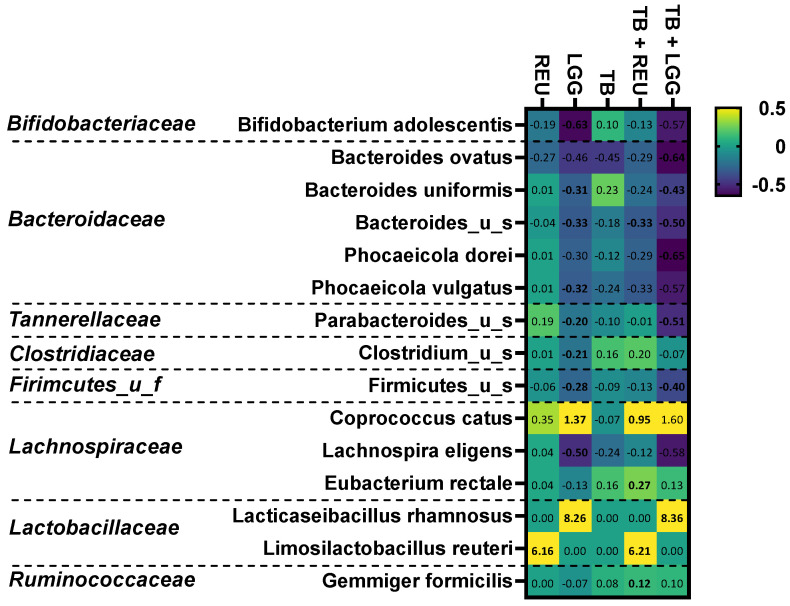
**The impact of two *Lactobacillaceae species* (LGG and REU), tributyrin oil (TB), and combinations thereof on microbial species** (belonging to specific families) that were significantly affected by any of the treatments compared to an untreated NSC (FDR = 0.20). Values are expressed as log_2_ (abundance treatment/abundance NSC), as averaged over simulations for six human adults using the SIFR^®^ technology platform. Samples were collected at 48 h after initiation of the colonic incubations. Significant differences are indicated in bold.

TB as such already impacted microbial composition, as TB significantly decreased *Proteobacteria* levels and significantly increased the *Firmicutes* phylum (Figures S3 and S4). Further, TB also tended to increase *Actinobacteria* due to a tendency towards higher *Bifidobacterium adolescentis* levels (Figure 5). Additionally, supplementation of LGG and REU as such already impacted microbial composition. At the phylum level, LGG and REU significantly increased *Firmicutes* levels (Figures S3 and S4), which was, in part, due to marked increases in *Limosilactobacillus reuteri* (NSC: <limit of detection (LOD), REU: 3.9 ± 0.3%) and *Lacticaseibacillus rhamnosus* (NSC: <LOD, REU: 16.6 ± 2.3%)*,* respectively (Figure 5). Further, in contrast to REU, LGG also significantly boosted *Coprococcus catus,* a lactate-consuming, butyrate-producing species, while decreasing the abundances of a series of species belonging to the *Actinobacteria*, *Bacteroidetes*, *Firmicutes,* and *Proteobacteria* phyla (Figure 5). It should be noted that the current analysis was based on relative abundances and not absolute levels. Lower relative abundances of several taxa (e.g., *B. adolescentis*) upon REU/LGG addition do not necessarily indicate a decreased absolute level of these taxa but could rather reflect the higher total number of bacteria upon REU/LGG addition. This makes the increase in relative abundances of *C. catus* even more striking.

**Figure 6:**
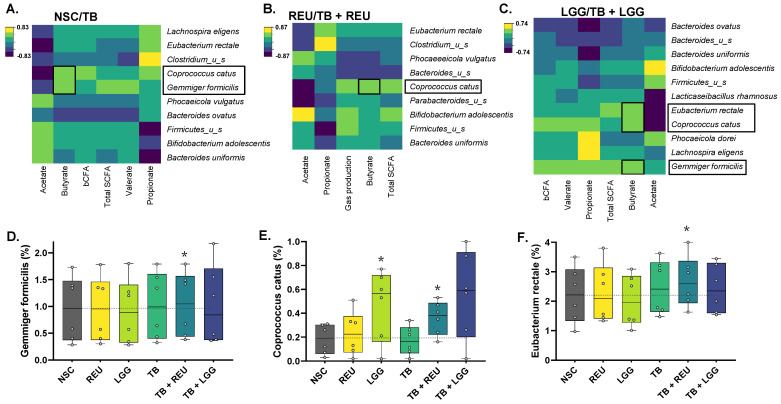
**Regularized Canonical Correlation Analysis (rCCA) highlights correlations between butyrate and compositional data at the species level**, focusing on (**A**) TB-treated samples, (**B**) REU-treated samples (with and without TB), and (**C**) LGG-treated samples (with and without TB). Bar plots of relative abundance of the three selected species involved in butyrate production according to the rCCA (**D**–**F**), across simulations for six healthy donors (n = 6). Statistically significant differences (FDR = 0.20) compared to the NSC is marked with an asterisk.

Interestingly, combinations of TB with REU/LGG significantly increased *Firmicutes* at the expense of *Bacteroidetes*, not only compared to the NSC and TB but also compared to REU/LGG alone (Figures S3 and S4), suggesting that LGG and REU specifically alters microbial composition in the presence of TB. At the species level, TB + REU significantly stimulated butyrate-producing species belonging to both the *Lachnospiraceae* (*Coprococcus catus* and *Eubacterium rectale*) and *Ruminococcaceae* (*Gemmiger formicilis*) families (Figure 5 and Figure 6D–F). This demonstrates the potential of TB + REU to shift microbial composition towards butyrate-producing species. A key contribution of *Coprococcus catus* to butyrate production upon TB + REU treatment was suggested by its correlation with butyrate levels (Figure 6B). 

Similarly to LGG alone, its combination with TB increased *Coprococcus catus* levels (Figure 5). This increase was, however, not significant given the larger interpersonal variation of this treatment effect (e.g., no increase for donor 5, in contrast to marked increases for donors 1/2) (Figure 6E). In samples containing LGG, butyrate levels were positively correlated with *Coprococcus catus*, *Eubacterium rectale,* and *Gemmiger formicilis* (Figure 6C).

## 3. Discussion

This study evaluated the potential to manage butyrate supply to the host via tributyrin oil (TB), whether or not co-supplemented with a *Lactobacillaceae* species (REU or LGG). The research question was addressed using the ex vivo SIFR^®^ technology, a recently developed gut model that, within 24–48 h, provides insights into gut microbiota modulation that are predictive for observations of repeated-intake clinical studies (down to species level resolution) [37]. In line with the aforementioned study, a high technical reproducibility, marked metabolite production, high microbial diversity, and, most importantly, sustained similarity between the original donor microbiota and untreated SIFR^®^ reactors at 48 h was observed during the current study. Such sustained similarity is fundamentally different from consistent biases observed for the current generation of in vitro gut models [26,27,28,29,30,31,32] and is the basis of classifying the application of SIFR^®^ technology as an ex vivo study, which is a study that uses an artificial environment outside the human body with minimum alteration of natural conditions. Further, owing to its high throughput, the SIFR^®^ technology enabled the inclusion of multiple test subjects in the study design. Interestingly, the differences in microbiota composition among the six human adults were mainly driven by different levels of *Prevotellaceae, Ruminococcaceae,* and *Bacteroidaceae,* families that have previously been identified as key taxa to stratify the human adult gut microbiota according to the concept of enterotypes [38]. This suggests that the six human adults covered a spectrum of microbial composition that can occur in vivo. Despite this heterogeneity at baseline, LGG and REU remained viable upon administration to the microbiota of each human adult and even slightly increased in abundance towards the end of the incubation. Interestingly, remarkably consistent treatment effects on butyrate were noted for TB and its combinations with REU and LGG.

First, when administered as such, TB consistently increased butyrate levels with 83 ± 6% of the theoretical butyrate content of TB, suggesting an efficient hydrolysis of TB to glycerol and butyrate. Further, dosing 1 g TB/L already mildly shaped microbial composition based on tendencies towards higher *Bifidobacterium adolescentis* levels and significantly decreased *Proteobacteria* levels. While the translation of the data can be questioned given the very different host physiologies, these results are in line with studies where common and grass carp were supplemented with TB [39,40]. Proteobacteria are gut commensals usually present in low numbers, whereas, under specific triggers, they can increase in number and become colitogenic microbes causing inflammatory responses [41,42]. Therefore, a reduction in *Proteobacteria* members could be an additional mechanism to generate health benefits upon TB administration beyond the highly consistent increase in butyrate levels across donors due to the direct release of butyrate from TB.

Co-administration of REU and LGG consistently increased butyrate levels up to 138 ± 11% and 126 ± 8% of the theoretical butyrate content of TB. The high consistency of this additional butyrate increase upon co-supplementation of REU/LGG is even more remarkable as REU/LGG are not able to produce butyrate themselves but require specific microbes from the indigenous microbiota to produce butyrate. Given the aforementioned heterogeneity of microbiota composition between human adults and the diversity of butyrate producers in the gut [43], the specific increase in *C. catus* by TB + LGG and particularly TB + REU suggests a strong complementarity between REU/LGG and *C. catus* in the presence of TB. While *Coprococcus catus* can convert lactate to propionate via the acrylate pathway [44], *C. catus* also has the enzymatic machinery to produce butyrate via the butyryl-CoA:acetate CoA-transferase route [19]. *C. catus* could thus thrive on lactate that can indeed be produced by LGG/REU from glycerol [45]. Nevertheless, other lactate-utilizing, butyrate-producing species such as *Anaerobutyricum hallii* were also consistently detected in the microbiota of each of the six donors at baseline levels (0.8 ± 0.1%) that even exceeded those of *C. catus* (0.4 ± 0.2%). While *A. hallii* strongly increased and correlated with butyrate production upon inulin and 2′FL treatment in previous SIFR^®^ studies [37], *A. hallii* was unaffected by TB + REU/LGG treatment in the current study, further highlighting the remarkable complementarity between *C. catus* and REU/LGG in the presence of TB. As a potential explanation, Sheridan et al. (2022) recently demonstrated that glucose partially repressed lactate utilization (*lct*) cluster expression in *A. soehngenii* (another lactate-consuming, butyrate-producing species), while such repression was not observed for *C. catus* [46]. It will be interesting to unravel the underlying mechanisms that could render *C. catus* more competitive compared to other lactate-utilizing butyrate producers in the presence of TB and REU/LGG.

In contrast to TB + LGG, the combination of TB with REU also significantly stimulated the butyrate-producing species *Eubacterium rectale* and *Gemmiger formicilis* while significantly increasing microbial diversity. A high microbial diversity is generally considered to contribute to ecosystem resilience after disturbance to the microbiome, and it has been reported to be generally higher in healthy compared to compromised subjects [47,48]. A unique effect of REU could potentially follow from its capability to convert glycerol not only to lactate but also to intermediate metabolites such as 1,3-propanediol (1,3-PDO) and 3-hydroxypropionate (3-HPA) [49,50,51,52]. 3-HPA, also known as reuterin, is a potent antimicrobial compound with inhibitory effects against multiple microorganisms, including *Escherichia, Salmonella, Shigella, Proteus, Pseudomonas, Clostridium*, and *Staphylococcus*, and to a lesser extent lactic acid bacteria [53]. Reuterin inhibits bacterial growth by affecting thiol groups and inducing oxidative stress [54]. The production of reuterin by REU could further regulate microbial composition by limiting the growth of reuterin-sensitive species, thus freeing ecological niches for other potentially butyrate-producing gut microbes, which could explain the more pronounced effect of TB + REU on butyrate and microbial diversity compared to TB + LGG.

While the present study demonstrated an interesting strategy to supply butyrate to the host via a combined direct (via hydrolysis of TB) and indirect (via cross-feeding on glycerol) butyrate stimulation, the next critical step is to demonstrate that these mechanisms can be translated to an in vivo setting. For this purpose, combined delivery of TB and viable cells of LGG/REU in a GIT region where the *Lactobacillaceae* species can be metabolically active is compulsory. While the proximal colon could be targeted, the distal small intestine could be a more appropriate landing platform for this synbiotic concept given the lower density of the indigenous microbiota in this region [55], thus allowing REU/LGG to preferentially ferment glycerol and prepare for cross-feeding interactions with butyrate producers. Another important aspect to obtain health benefits is to optimize the test dose. As 1 g TB oil resulted in the direct release of around 4 mmol butyrate and an additional production of 2–3 mmol in the presence of LGG-REU, a total amount of around 6–7 mmol could be delivered from 1 g TB oil. To put these values in perspective, daily total SCFA production can be considered: assuming a daily intake of 20 g fiber/day in a healthy human adult, total daily SCFA production is in the range of 200 mmol/day [56]. If butyrate represents 20% of total SCFA levels, 40 mmol butyrate would be produced per day due to fiber intake. This would suggest that consumption of 1 g TB per day could increase butyrate supply to healthy human adults with 10% (TB alone) or even 15% upon co-supplementation with LGG/REU. Considering that the fiber intake is reported to be well below 20 g/d in most countries [57,58], TB or symbiotic combinations are promising strategies to increase health-promoting butyrate in the human gut. There is, however, growing awareness that SCFA production upon fiber intake is prone to marked interpersonal differences. Further, when considering individuals with low fiber intake or subjects with a microbiota depleted in butyrate producers [59,60,61,62,63], daily butyrate production could be much lower than 40 mmol/day, in which case 6–7 mmol butyrate could represent a significantly larger fraction of the basal daily butyrate production.

While a key advantage of the SIFR^®^ technology is the absence of a host component, enabling unique insights into metabolite production and microbial composition that are hard to obtain in vivo, a related drawback is that findings of such ex vivo studies should be regarded as complementary to in vivo studies, rather than as a potential replacement of clinical studies. Despite the high predictivity of the SIFR^®^ technology for clinical findings [37], clinical studies are required to demonstrate potential health benefits for the host upon TB and LGG/REU co-supplementation.

In conclusion, both the direct butyrate stimulation (via hydrolysis of TB) and additional indirect butyrate increase (via REU/LGG-mediated cross-feeding on glycerol) were remarkably consistent across the six human adults tested ex vivo. Especially the latter was remarkable, as it involved the contribution of a specific species of the indigenous human gut microbiota, i.e., *Coprococcus catus.* This high consistency contrasts with the large interpersonal differences in butyrate production that are often observed upon prebiotic treatment. Combining TB with LGG and especially REU is thus a promising strategy to consistently supply butyrate to the host, potentially resulting in more predictable health benefits. While the study focused on potential health benefits due to butyrate supply, probiotic administration as such could also contribute to additional health benefits [35]. 

## 4. Materials and Methods

### 4.1. Test Products: TB, LGG, and REU

Tributyrin (TB) oil (≥95% purity) was obtained from NutriScience Innovations (Milford, United States of America) and tested at 1000 mg/L.

*Lacticaseibacillus rhamnosus GG* ATCC 53103 (LGG) and *Limosilactobacillus reuteri* ATCC 53608 (REU) were obtained from the Belgian Coordinated Collections of Microorganisms-Laboratory for Microbiology Ghent (BCCM-LMG, Ghent, Belgium). LGG and REU were grown under anaerobic conditions for 24 h at 37 °C. A first subculture was pre-pared on a selective solid growth medium (LAMVAB agar [64]). Subsequently, cells derived from a single colony were grown in MRS broth under anaerobic conditions for 24 h at 37 °C, after which the strain was stored at −80 °C in MRS, with 20% (*vol*/*vol*) of glycerol as a cryoprotectant. Prior to its use, the cryopreserved strain was inoculated in MRS broth and grown under anaerobic conditions for 24 h at 37 °C. Bacterial cells were centrifuged during 5′ at 3000× *g* and resuspended in anaerobic PBS prior to inoculation in the SIFR^®^ reactors at a final density of around 5 × 10^7^ CFU/mL. 

### 4.2. SIFR^®^ Technology

The SIFR^®^ technology was recently validated and enables the study of the human gut microbiota in a highly biorelevant manner for multiple test conditions (both treatments and test subjects) [37]. Briefly, individual bioreactors were processed in parallel in a bioreactor management device (Cryptobiotix, Ghent, Belgium). Each bioreactor contained 5 mL of nutritional medium-fecal inoculum blend supplemented with test products, then sealed individually, before being rendered anaerobic. Blend M0003 was used for the preparation of the nutritional medium (Cryptobiotix, Ghent, Belgium). After preparation, bioreactors were incubated under continuous agitation (140 rpm) at 37 °C for 48 h (MaxQ 6000, Thermo Scientific, Thermo Fisher Scientific, Merelbeke, Belgium). Upon gas pressure measurement in the headspace, liquid samples were collected for subsequent analysis.

Fresh fecal samples were collected according to a procedure approved by Ethics Committee of the University Hospital Ghent (reference number BC-09977). This involved participants signing an informed consent to donate their fecal sample for the current study. The selection criteria for the 6 donors used were as follows: age 25–65, no antibiotic use in the past 3 months, no gastro-intestinal disorders (cancer, ulcers, IBD), no use of probiotic, non-smoking, alcohol consumption < 3 units/d, and BMI < 30. For this specific study, four male and two female donors were tested with an average age of 28.8 ± 1.6 years.

### 4.3. Study Design

**Figure 7:**
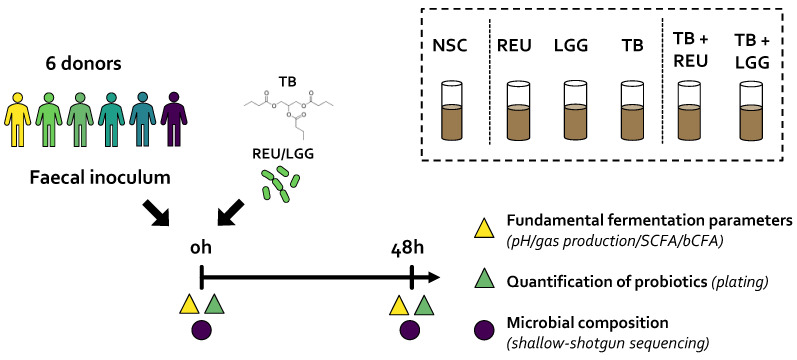
**Experimental design using SIFR^®^ technology platform to test the impact of tributyrin oil (TB), *Lactobacillaceae species* (REU and LGG) and combinations thereof on the human gut microbiota**. Fecal samples from 6 healthy donors were cultivated ex vivo to assess the impact on the microbial metabolite production and composition. REU = *Limosilactobacillus reuteri* ATCC 53608; LGG = *Lacticaseibacillus rhamnosus* GG ATCC 53103; SCFA = short chain fatty acids; bCFA = branched fatty acids.

Six study arms were tested for each of the six fecal microbiota: (i) NSC containing background medium and fecal microbiota without products, (ii) LGG, (iii) REU, (iv) TB, (v) TB + LGG, and (vi) TB + REU (Figure 7). The NSC was tested in technical triplicate to confirm the previously demonstrated high technical reproducibility of the SIFR^®^ technology [37], which allows focusing on biological replicates rather than technical replicates, as was also the case in the current study, where all treatments were tested for six independent donors (as biological replicates). Samples were collected at 0 h and 48 h for fundamental fermentation parameters (pH, gas, SCFA and bCFA) and microbial composition (shallow shotgun sequencing) (Figure 6). LGG and REU were additionally quantified via culture-based enumeration at 0 h and 48 h. 

The untreated no-substrate control (NSC) incubations were additionally run in n = 3 for each donor (n = 6). Coefficients of variation of pH, gas production, and the three main SCFA (acetate, propionate, and butyrate), were on average as low as 1.74%, which comprises all variation from medium and inoculum preparation up to sample analysis. Such high reproducibility renders the SIFR^®^ technology sensitive to unraveling the impact of test ingredients on the complex gut microbiota.

### 4.4. Fundamental Fermentation Parameters

SCFA (acetate, propionate, butyrate, and valerate) and branched-chain fatty acids (bCFA; sum of isobutyrate, isocaproate, and isovalerate) were determined via GC with flame ionization detection (FID) (Trace 1300, Thermo Fisher Scientific, Merelbeke, Belgium), upon diethyl ether extraction. Briefly, 0.5 mL samples were diluted in distilled water (1:3) and acidified with 0.5 mL 48% sulfuric acid, after which an excess of sodium chloride was added along with 0.2 mL internal standard (2-methylhexanoic acid) and 2 mL diethyl ether. Upon homogenization and subsequent separation of the water and diethyl ether layer, diethyl ether extracts were analyzed on the GC-FID using nitrogen gas as carrier and makeup gas as previously described [65]. pH was measured using an electrode (Hannah Instruments Edge HI2002, Temse, Belgium).

### 4.5. Selective Enumeration of Lactobacillaceae species (LGG and REU)

At 0 h and 48 h, samples were collected from different rectors and viable counts of *Lactobacillaceae species* were determined by making dilution series in PBS, followed by selective enumeration on LAMVAB agar [64], and incubated aerobically (LGG) or anaerobically (REU) during 48 h.

### 4.6. Microbiota Phylogenetic Analysis via Shallow Shotgun Sequencing

Initially, a bacterial cell pellet was obtained by centrifugation of 1 mL sample for 5 min at 9000× *g*. DNA was extracted via the SPINeasy DNA Kit for Soil (MP Biomedicals, Eschwege, Germany), according to manufacturer’s instructions. Subsequently, DNA libraries were prepared using the Nextera XT DNA Library Preparation Kit (Illumina, San Diego, CA, USA) and IDT Unique Dual Indexes with total DNA input of 1 ng. Genomic DNA was fragmented using a proportional amount of Illumina Nextera XT fragmentation enzyme. Unique dual indexes were added to each sample followed by 12 cycles of PCR to construct libraries. DNA libraries were purified using AMpure magnetic Beads (Beckman Coulter, Brea, CA, USA), eluted in QIAGEN EB buffer, quantified using a Qubit 4 fluorometer and a Qubit dsDNA HS Assay Kit, and sequenced on an Illumina Nextseq 2000 platform 2 × 150 bp. Unassembled sequencing reads were converted to relative abundances (%) using the CosmosID-HUB Microbiome Platform (CosmosID Inc., Germantown, MD, USA) [66,67]. 

### 4.7. Data Analyses

All univariate and multivariate analyses were performed by GraphPad Prism (v9.3.1; www.graphpad.com accessed on 26 December 2022), while Regularized Canonical Correlation Analysis (rCCA) was executed using the mixOmics package with the shrinkage method for estimation of penalization parameters (version 6.16.3) in R (4.1.1; www.r-project.org; accessed on 26 December 2022) [68]. Treatment effects were assessed using repeated measures ANOVA analysis (based on paired testing) and *p*-values were corrected with Benjamini–Hochberg [69] (FDR = 0.05). For the analysis of microbial composition, a threshold was set in order to retain the 100 most abundant species in the analysis, to avoid excessive *p*-value corrections. 

## Data Availability

The datasets generated during and/or analyzed during the current study are available from the corresponding author with approval from funding source upon reasonable request.

## Supplementary Materials

The following supporting information can be downloaded at https://www.mdpi.com/article/10.3390/ijms24065859/s1.
