# Supplementary material for: Lacticaseibacillus rhamnosus ATCC 53103 and Limosilactobacillus reuteri ATCC 53608 Synergistically Boost Butyrate Levels upon Tributyrin Administration Ex Vivo
Source: Int J Mol Sci. 2023 Mar 20;24(6):5859. doi: 10.3390/ijms24065859 (PMC10054277; doi:10.3390/ijms24065859)
Supplement: Supplementary file 1 [file ijms-24-05859-s001.zip › ijms-2153253-supplementary.pdf]

International Journal of
Molecular Sciences

MDPI

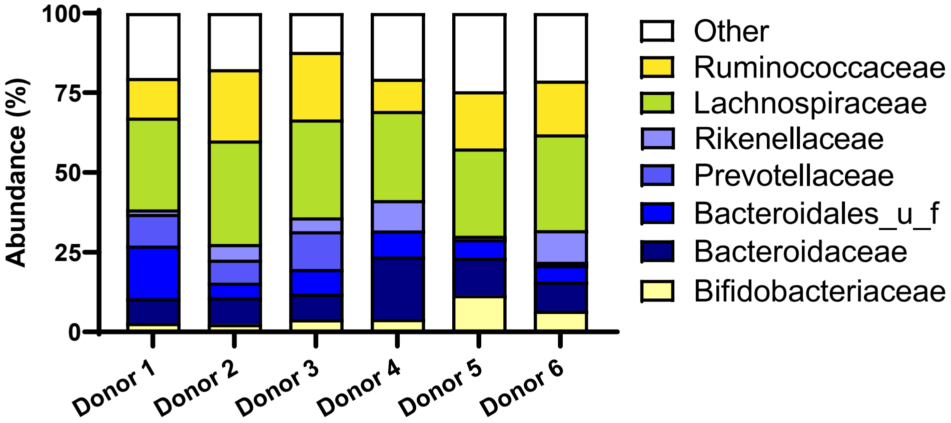

Figure S1. Distinct interpersonal differences in microbial composition were observed for the 6 human adults. Abundances (%) of the families that were mainly responsible for differences across samples in Figure 1.

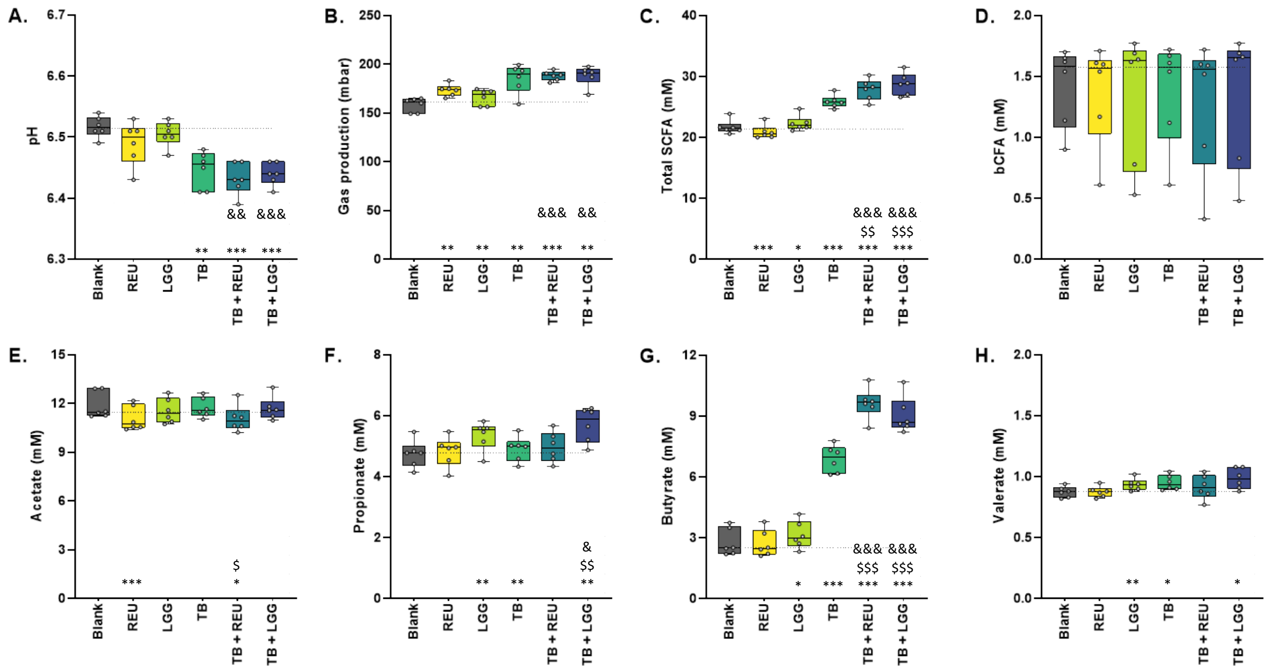

Figure S2. The impact of two *Lactobacillaceae* species (LGG and REU), tributyrin oil (TB) and combinations thereof on fundamental fermentation parameters: pH (A), gas production (B), total SCFA (C), bCFA (D), acetate (E), propionate (F), butyrate (G) and valerate levels (H), for simulations of 6 human adults using the SIFR® technology (n = 6). Samples were collected after 48h incubation. The lines in the box plots are shown at the median value of the NSC. Statistical differences compared to the NSC are indicated with * (0.01 < $p_{adjusted}$ < 0.05), ** (0.001 < $p_{adjusted}$ < 0.01) or *** ($p_{adjusted}$ < 0.001), while differences between TB+REU/TB+LGG and TB are indicated with $/$$/$$$. Differences between TB+REU/TB+LGG and the respective probiotic (REU/LGG) are indicated with &/&&/&&&. bCFA = branched fatty acids; NSC = no substrate control; REU = *Limosilactobacillus reuteri*; LGG = *Lacticaseibacillus rhamnosus*.

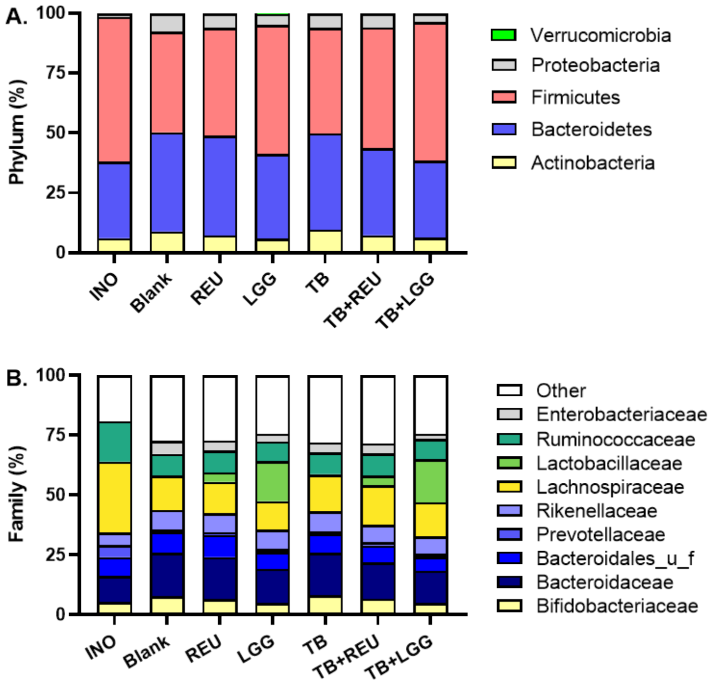

Figure S3. Impact of two *Lactobacillaceae* species (LGG and REU), tributyrin oil (TB) and combinations thereof on microbial composition at phylum (A) and family level (B) for the most abundant taxa, as averaged across simulations for 6 human adults using the SIFR® technology (n = 6). Samples were collected at 0h (INO) and after 48h of simulated colonic incubations.

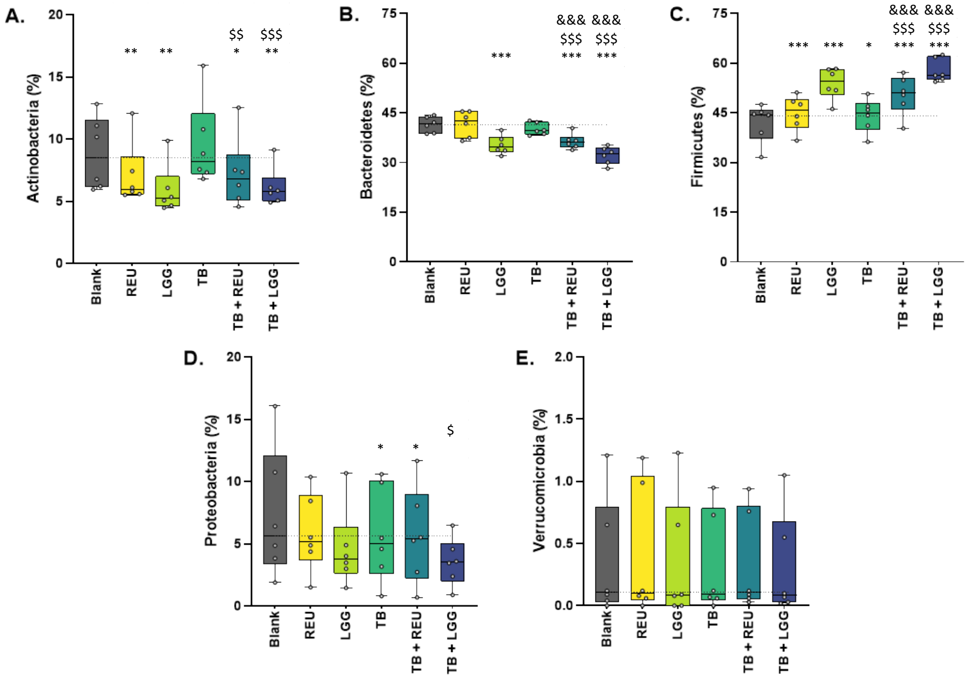

Figure S4. Impact of two *Lactobacillaceae* species (LGG and REU), tributyrin oil (TB) and combinations thereof on microbial composition at phylum level (%): Actinobacteria (A), Bacteroidetes (B), Firmicutes (C), Proteobacteria (D) and Verrucomicrobia (E). Samples were collected after 48h incubation. The lines in the box plots are shown at the median value of the NSC. Statistical differences compared to the NSC are indicated with * ($0.10 < p_{adjusted} < 0.20$), ** ($0.05 < p_{adjusted} < 0.10$) or *** ($p_{adjusted} < 0.05$), while differences between TB+REU/TB+LGG and TB are indicated with $/$$/$$$. Differences between TB+REU/TB+LGG and the respective probiotic (REU/LGG) are indicated with &/&&/&&&. bCFA = branched fatty acids; NSC = no substrate control; REU = *Limosilactobacillus reuteri*; LGG = *Lacticaseibacillus rhamnosus.*
